# Circulating Cathelicidin Concentrations in a Cohort of Healthy Children: Influence of Age, Body Composition, Gender and Vitamin D Status

**DOI:** 10.1371/journal.pone.0152711

**Published:** 2016-05-06

**Authors:** Taylor M. Stukes, Judith R. Shary, Wei Wei, Myla D. Ebeling, Kaleena B. Dezsi, Frank S. Shary, Nina E. Forestieri, Bruce W. Hollis, Carol L. Wagner

**Affiliations:** 1 Department of Pediatrics, Medical University of South Carolina, Charleston, South Carolina, United States of America; 2 Department of Public Health Sciences, Medical University of South Carolina, Charleston, South Carolina, United States of America; University of Florida, UNITED STATES

## Abstract

Cathelicidin is an antimicrobial peptide whose circulating levels are related to vitamin D status in adults. This study sought to determine if circulating cathelicidin concentrations in healthy children are related to the age of the child, body composition and vitamin D status at birth and at the time of the study visit. Blood samples were obtained during yearly visits from 133 children, ages 2–7, whose mothers had participated in a pregnancy vitamin D supplementation RCT. Radioimmunoassay and ELISA were performed to analyze 25(OH)D and cathelicidin, respectively. Statistical analyses compared cathelicidin concentrations with concentrations of 25(OH)D at various time points (maternal levels throughout pregnancy, at birth, and child’s current level); and with race/ethnicity, age, gender, BMI, percent fat, and frequency of infections using Student’s t-test, χ^2^, Wilcoxon ranked-sum analysis, and multivariate regression. The cohort’s median cathelicidin concentration was 28.1 ng/mL (range: 5.6–3368.6) and did not correlate with 25(OH)D, but was positively correlated with advancing age (ρ = 0.236 & p = 0.005, respectively). Forty patients evaluated at two visits showed an increase of 24.0 ng/mL in cathelicidin from the first visit to the next (p<0.0001). Increased age and male gender were correlated with increased cathelicidin when controlling for race/ethnicity, percent fat, and child’s current 25(OH)D concentration (p = 0.028 & p = 0.047, respectively). This study demonstrated that as children age, the concentration of cathelicidin increases. Furthermore, male gender was significantly associated with increased cathelicidin concentrations. The lack of association between vitamin D status and cathelicidin in this study may be due to the narrow range in observed 25(OH)D values and warrants additional studies for further observation.

## Introduction

Cathelicidin (Human LL-37), activated by n-terminal cleavage of the propeptide hCAP18, serves as a cytokine in the immune system as well as an antimicrobial peptide with its ability to form an α-helix in aqueous solution that disrupts the lipid membranes of invading organisms and results in their destruction [1–4]. Primarily utilized by monocytes, macrophages, mucosal epithelial cells, and keratinocytes as part of the innate immune system, these cells release cathelicidin along with other cytokines and signal molecules as a first line of defense [5]. Previous studies have focused on cathelicidin and vitamin D concentrations in patient populations affected by various illnesses or diseases [6], such as tuberculosis [7], pneumonia [8], sepsis [9], HIV [10–12], and end-stage renal disease hemodialysis [13], as well as healthy adult populations [14–19]. Additionally, while one study examined the levels of maternal and neonatal vitamin D and cathelicidin concentrations in healthy children at delivery, there is a lack of research to determine correlations between vitamin D status and cathelicidin among healthy children over time [20], as well as which factors such as age of the child, gender, and body composition may independently impact cathelicidin concentrations.

To address this gap in the literature, the aim of this follow-up study was to determine if maternal vitamin D status during pregnancy and/or a child’s current vitamin D status affect the circulating concentration of cathelicidin in healthy children years after delivery. A second aim was to determine if the concentration of cathelicidin and vitamin D correlate with the number of infections a child experiences during childhood and as a function of age. Offspring of mothers enrolled in an NICHD pregnancy vitamin D supplementation trial (results of which were previously published [21, 22]) were seen in follow-up from ages 2–7 years during a three-year study period. It was hypothesized that (1) total current circulating 25(OH)D would be positively correlated with cathelicidin in children; (2) children with higher plasma cathelicidin concentrations would have had fewer infections than children with lower cathelicidin concentrations; and (3) cathelicidin concentration would increase with advancing age. The results of this study to address these hypotheses are presented here.

## Materials and Methods

### Subjects

The offspring of mothers who had participated in an NICHD, IRB-approved vitamin D supplementation study during pregnancy (HR#10727; CTRC protocol #670; n = 350) were invited back for yearly follow-up beginning at 2 years of age. The study had recruited women from 2004–2009. Mothers were randomized to receive 400, 2000, or 4000 IU vitamin D_3_/day beginning at 12 to 16 weeks of gestation and continuing through delivery [22, 23]. Women who had participated in the NICHD trial provided their written informed consent. The children of mothers participating in the NICHD trial were then seen at yearly follow-up visits for three years between the ages of 2 and 7 as part of an IRB-approved follow-up study funded by the Thrasher Research Fund (HR# 19641, CTRC protocol 870). Written informed consent was obtained from the parents/guardians on behalf of the children enrolled in this study. All clinical investigation was conducted according to the Declaration of Helsinki. There were 194 children who participated in the follow-up study (55% of the original cohort), but blood samples were obtained from 133 of those children.

### Study Protocol

#### Health History Information

During the yearly visits, 5-mL of whole blood were obtained by venous puncture, and the child’s health status, medication usage, and hospitalization history were assessed by both standardized questionnaires completed by a parent of each child and the child’s medical records. Health and dietary information were obtained from parental report and confirmed by medical records from the primary care physician or the hospital. The Block Children’s Food Frequency questionnaire was administered at each yearly visit [24]. Infections were defined as discrete events that were documented in the medical record and/or the primary health care record. If there were two types of infection during a single visit to the physician or hospital, that event was counted as a single event.

#### Anthropometric Measures

Height (cm) was measured with a Harpenden stadiometer (Holtain, Limited, Crosswell, UK) and weight (kg) was measured using the Healthometer ProPlus scale, (Welch Allyn, Inc., Skaneateles Falls, NY). Body fat percentage of each child was determined by DEXA and recorded. All whole body DEXA scans were done by the same technologist on a Hologic Discovery A densitometer (Bedford, MA, USA). Inter- and intra-assay coefficients of variation for the hip and spine phantoms were 1% or less.

#### Blood Measures

Total circulating serum 25(OH)D concentration was measured in duplicate within the month the blood sample was obtained using radioimmunoassay (RIA; DiaSorin, Stillwater, MN). Plasma cathelicidin was measured from the children’s samples by ELISA (Hycult Biotech, the Netherlands), in which each sample was diluted 24 times and analyzed in duplicate. Samples with concentrations above the standard curve upper limit were diluted further due to their higher cathelicidin concentration. Data were accepted with a coefficient of variance (CV) of <10%. The detection limit of the human cathelicidin ELISA was 0.14 ng/mL.

### Statistical Analysis

Analysis was completed using the cathelicidin concentration values obtained from each child’s yearly follow-up visit. Spearman correlations (ρ) were used to assess the association between each child’s cathelicidin concentration and the 25(OH)D concentrations obtained at various time points: (1) at the corresponding follow-up visit; (2) at delivery; (3) maternal concentration of 25(OH)D throughout pregnancy (using the area under the curve from visit 3 to visit 7); and (4) maternal concentration of 25(OH)D within one month of delivery. Each child’s cathelicidin concentration also was compared to the frequency of infections each child had diagnosed within the last year, and to the age of the child. A *post-hoc* analysis was conducted to determine if BMI and percent fat had an effect on cathelicidin concentration. In the 40 children in whom repeat yearly cathelicidin concentrations were available, a Wilcoxon signed rank test of paired samples was performed. Four multivariable regressions were performed to assess whether any significant associations existed between the natural logarithm of each subject’s cathelicidin concentration and each of the following vitamin D measurements: (1) child’s current 25(OH)D at time of follow-up, (2) child’s 25(OH)D at birth, (3) maternal 25(OH)D throughout pregnancy (Visits 3 to 7 as area under the curve), and (4) maternal 25(OH)D one month before delivery; all analyses controlled for race/ethnicity, age, gender, and percent fat. Using the Institute of Medicine’s definition of sufficiency (total circulating 25(OH)D ≥20 ng/mL (50 nmol/L) [25], the cohort also was divided into two groups based on child’s current vitamin D status, with those with 25(OH)D concentrations <20 ng/mL (50.0 nmol/L) defined as deficient. Statistical analyses were performed using SAS 9.3. Bivariate analysis was performed using χ^2^; mean continuous variables were analyzed using Student’s t-test; median values were analyzed using Wilcoxon ranked-sum analysis; and multivariate analyses were analyzed using linear regression. The size of this study’s cohort provided us with 80% power to detect correlations as small as 0.24.

## Results

### Cohort

While 194 children had participated in the follow-up study (55% of the original cohort participating in the vitamin D pregnancy study), 133 (38%) had a blood sample obtained at the time of the study visit and thus, were able to have cathelicidin measured.

**Table 1** shows the demographics and clinical characteristics of the 133 children participants in this study with measurement of circulating cathelicidin. The cohort, consisting of 46.6% females and 53.4% males, included 41 Blacks, 38 Whites, and 54 Hispanics. The children’s mean current 25(OH)D at the time of the follow-up visit was 27.2 ng/mL (range 8.30–61.5 ng/mL; 68.3 nmol/L). Of the children in the cohort, 20.3% were found to be vitamin D deficient, as defined by a 25(OH)D concentration <20 ng/mL.

**Table 1:** Child Sociodemographics and Clinical Characteristics.

Characteristic	Value
	n = 133
Race/Ethnicity [n (%)]	
Black	41 (30.8)
White	38 (28.6)
Hispanic	54 (40.6)
Gender [n (%)]	
Female	62 (46.6)
Male	71 (53.4)
Age^1^ (years)	4.7 ± 1.2 (2–7)
BMI^1^	16.1 ± 2.5 (12.1–28.8)
% Fat^1^	25.5 ± 6.1 (14.6–42.9)
No. of Infections in the last year [median (range)]	1.0 (0.0–5.0)
Child’s daily vitamin D intake at time of visit (IU/day)^1^	205.3 ± 109.8 (17.1–512.5)
Child's 25(OH)D at time of visit (ng/mL)^1^	27.2 ± 9.4 (8.3–61.5)
Child's vitamin D status at time of visit	
Sufficient (≥20 ng/mL) [n (%)]	106 (79.7)
Deficient (<20 ng/mL) [n (%)]	27 (20.3)
Total Circulating 25(OH)D (ng/mL) at birth^1^	22.8 ± 9.8 (3.6–47.8)
Maternal 25(OH)D (ng/mL) within 1 month prior to delivery^1^	39.2 ± 15.0 (10.0–78.6)
Maternal 25(OH)D area under curve, pregnancy visits 3–7^1^	153.1 ± 44.6 (50.1–251.4)

^1^ mean±STD (range)

### Study Outcomes

The median cathelicidin concentration for the entire cohort was 28.1 ng/mL, ranging from 5.6 to 3368.6 ng/mL. Three subjects had cathelicidin concentrations that were more than two times greater than the next lowest concentration. These subjects had cathelicidin values of 737.5, 2000.4, and 3368.6 ng/mL, while the next highest value was 195.6 ng/mL. The subjects with the three highest cathelicidin concentration values had characteristics including 25(OH)D, BMI, percent fat, race/ethnicity, and age, which were comparable to the corresponding variable represented in the entire cohort. Therefore, these three subjects were included in the analysis. Of note, one of these children was obese, one had seasonal allergies treated with a long-term steroid nasal spray, and the third had a history of febrile seizures.

Maternal 25(OH)D throughout pregnancy was calculated using the area under the curve of 25(OH)D concentration values from pregnancy visits 3–7. White mothers were found to have a significantly higher 25(OH)D concentration (45.8 ng/mL; 114.5 nmol/L) than both Black (34.8 ng/mL; 87.0 nmol/L) and Hispanic mothers (38.0 ng/mL; 95.0 nmol/L) throughout pregnancy (p≤0.0001 and ≤0.0001, respectively). Black and Hispanic mothers were found to have a statistically significant difference in 25(OH)D concentrations throughout pregnancy as well (p≤0.0001).

A similar pattern was demonstrated in children’s 25(OH)D at the time of follow-up visit. White children had a mean 25(OH)D concentration of 35.1 ng/mL (87.8 nmol/L) at the follow-up visit, which was also found to be significantly higher than both Black and Hispanic children with mean 25(OH)D concentrations of 22.5 ng/mL (56.3 nmol/L) and 25.4 ng/mL (63.5 nmol/L), respectively (p≤0.0001 and ≤0.0001, respectively). Black and Hispanic children also had significantly different 25(OH)D concentrations at the time of follow-up visit (p = 0.023). Despite differences in vitamin D status, there were no significant associations between cathelicidin, race/ethnicity and vitamin D status (see **Table 2**). Cathelicidin concentrations were not significantly different among (or between) the three racial/ethnic groups (p = 0.999).

**Table 2:** Bivariate Analysis between Cathelicidin and Categorical Variables.

	Cathelicidin (ng/mL)	
Characteristic	Median (IQR)^1^	p-value
Race/Ethnicity		0.999
Black	26.8 (23.3, 41.6)	
White	27.7 (18.0, 51.9)	
Hispanic	30.2 (20.2, 39.0)	
Vitamin D status at Follow-up Visit		0.78
Deficient (<20 ng/mL)	27.4 (19.8, 35.9)	
Sufficient (≥20 ng/mL)	29.6 (20.2, 43.2)	

^1^ IQR represents 25th and 75th percentile

No significant overall correlation existed between the children’s cathelicidin concentration and the children’s 25(OH)D concentration at the time of follow-up visit, nor did one exist when the cohort was stratified by race/ethnicity. In addition, there were no significant correlations found between concentration of cathelicidin and the child’s 25(OH)D concentration at birth, maternal 25(OH)D within one month of delivery, or maternal 25(OH)D area under the curve from pregnancy visits 3–7. Neither BMI, nor percent fat were found to correlate with cathelicidin concentration.

**Fig 1:**
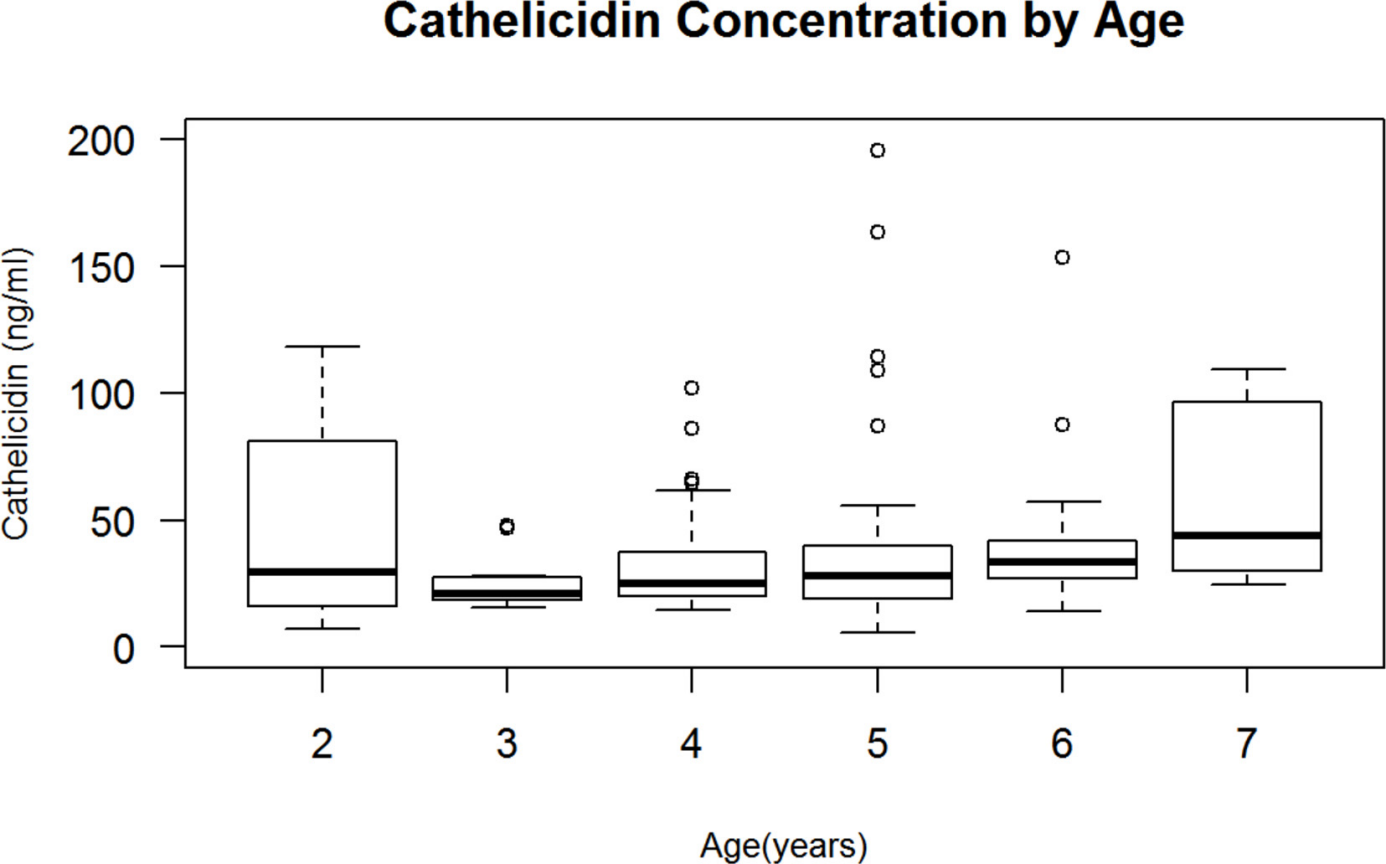
Cathelicidin Concentration by Age. There was a significant positive correlation between age and cathelicidin concentration (ρ = 0.236, p = 0.005); ρ value represents Spearman correlation. The upper and lower edges of the box represent the 75^th^ percentile and the 25^th^ percentile of cathelicidin concentration, respectively, measured by ELISA and reported in ng/mL.

Within the entire cohort, age of the child and cathelicidin concentration demonstrated a positive correlation (ρ = 0.236, p = 0.005). As the age of the child increased, the concentration of cathelicidin also increased (**Fig 1**). When the data were stratified by gender in *post-hoc* analyses, only the male population continued to demonstrate this correlation (ρ = 0.292, p = 0.014), while females did not (ρ = 0.103, p = 0.425). Although not statistically significant, a slightly negative trend of increased cathelicidin correlated with a decreased frequency of diagnosed infections within the last year (ρ = -0.147, p = 0.092).

When dividing the cohort by vitamin D sufficiency status for additional analyses, vitamin D deficiency was defined as <20 ng/mL (<50 nmol/L)). In vitamin D deficient children, there were no statistically significant differences between cathelicidin concentration among the races/ethnicities or by gender. Cathelicidin concentration in the deficient group did not correlate with percent fat, BMI, maternal 25(OH)D within one month prior to delivery, maternal 25(OH)D throughout pregnancy, child’s 25(OH)D at delivery, or child’s current 25(OH)D concentration. When comparing cathelicidin concentrations of children who were deficient with those who were sufficient, (25(OH)D concentrations ≥20 ng/mL), there were no statistically significant differences. In the group of children with a total circulating 25(OH)D concentration ≥20 ng /mL, cathelicidin concentration was significantly positively associated with age (ρ = 0.373, p = 0.0002). Within this same group, cathelicidin concentration was negatively associated with the number of documented infections a child experienced in the last year (ρ = -0.205, p = 0.047).

**Fig 2:**
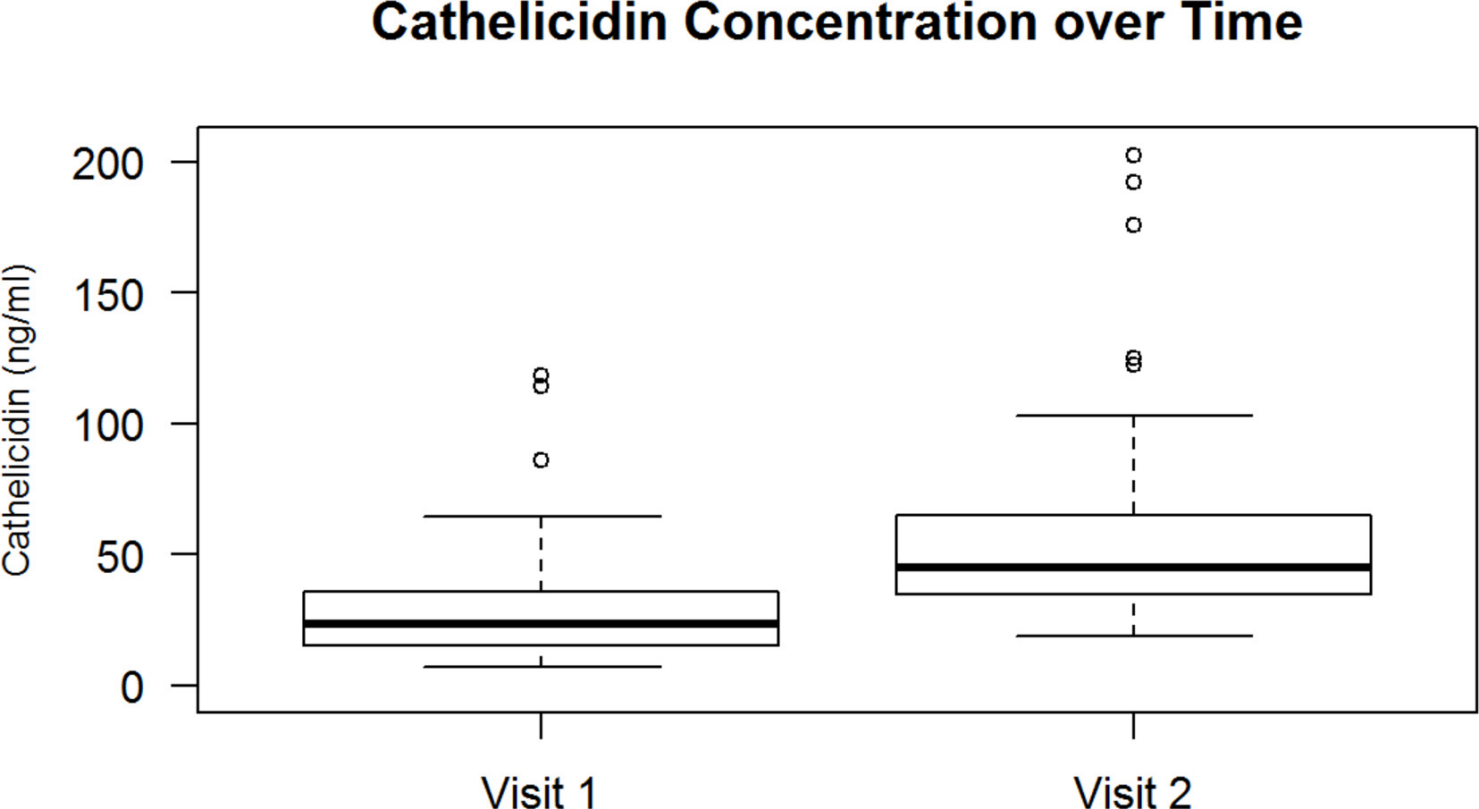
Change of Cathelicidin Concentration over Time. In a subset of 40 children with two consecutive visits roughly twelve months apart, the median increase in cathelicidin concentration from the first visit to the second visit was 24.0 ng/mL (ranging from -1.9 ng/mL to +223.4ng/mL, p≤0.0001); ρ value represents Spearman correlation. The upper and lower edges of the box represent the 75^th^ percentile and the 25^th^ percentile of cathelicidin concentration, respectively, reported in ng/mL.

Forty participants in this study had plasma samples collected at two subsequent yearly visits, spaced roughly twelve months apart. Among these subjects, their initial mean total circulating 25(OH)D concentration was 34.8 ng/mL (SD 13.0 ng/mL) and approximately 12 months later the mean concentration was 28.9 ng/mL (SD 9.2 ng/mL), which was not a clinically significant or statistically significant difference. Their median increase in cathelicidin from the first visit to the second visit was 24.0 ng/mL (ranging from -1.9 ng/mL to +223.4 ng/mL, p≤0.0001) (**Fig** 2).

**Table 3** summarizes the results of the 4 multivariable linear regression models assessing factors associated with the natural logarithm of the cathelicidin concentration. When adjusting for the other variables in the models, no significant relationship between any of the 25(OH)D measurements and cathelicidin was found. In each of the models, however, the child’s age at their first follow-up visit was significantly and independently positively correlated with cathelicidin. The beta estimate indicates how much the log cathelicidin concentration is predicted to increase with a 1-unit change in the independent variable, and exponentiating the beta value provides a fold-increase estimate. Thus, Model 1 indicates that every 1-year increase in a child’s age is associated with a 0.145 unit increase in their log cathelicidin concentration, equivalent to a e^0.145^ = 1.2-fold increase from 1 year to the next. In Models 1 and 4), higher cathelicidin concentrations were significantly associated with male gender (beta = 0.318, p = 0.047), indicating that after adjusting for the other variables in the model, males’ cathelicidin levels were 1.4 times higher than females’. In Models 1 and 4, there was also a trend noted with the child’s percent fat being positively associated with cathelicidin (beta = 0.028, p = 0.052 and beta = 0.025, p = 0.087).

**Table 3:** Multivariable Linear Regression Models Assessing Factors Associated with Natural Logarithm for Cathelicidin.

**Model 1**			
Independent Variables	Beta	SE^1^	p value
Race/Ethnicity			
Black	-0.039	0.23	0.864
White	---	---	---
Hispanic	-0.149	0.200	0.459
Gender			
Female	---	---	---
Male	0.318	0.16	**0.047**
Age	0.145	0.065	**0.028**
Percent Fat	0.028	0.014	0.052
Child's current 25(OH)D	-0.005	0.01	0.598
**Model 2**			
Independent Variables	Beta	SE	p value
Race/Ethnicity			
Black	0.035	0.21	0.868
White	---	---	---
Hispanic	-0.149	0.21	0.470
Gender			
Female	---	---	---
Male	0.275	0.17	0.104
Age	0.212	0.07	**0.004**
Percent Fat	0.025	0.02	0.087
Child's 25(OH)D at birth	-0.003	0.01	0.748
**Model 3**			
Independent Variables	Beta	SE	p value
Race/Ethnicity			
Black	0.041	0.18	0.821
White	---	---	---
Hispanic	-0.122	0.16	0.458
Gender			
Female	---	---	---
Male	0.091	0.14	0.507
Age	0.155	0.07	**0.023**
Percent Fat	0.012	0.01	0.359
Maternal 25(OH)D throughout pregnancy	<-0.0001	0	0.999
**Model 4**			
Independent Variables	Beta	SE	p value
Race/Ethnicity			
Black	0.046	0.199	0.816
White	---	---	---
Hispanic	-0.080	0.185	0.669
Gender			
Female	---	---	---
Male	0.318	0.158	**0.047**
Age	0.151	0.064	**0.021**
Percent Fat	0.026	0.01	0.069
Maternal 25(OH)D 1-month before delivery	0.003	0.01	0.606

^1^ SE represents Standard Error

## Discussion

In this study of healthy children whose mothers had participated in a vitamin D supplementation trial during pregnancy, despite differences in both maternal and childhood vitamin D status, there were no significant differences in cathelicidin concentration between the three racial/ethnic groups studied. A positive correlation between age and cathelicidin and between gender and cathelicidin were noted, with higher concentrations in male children.

Regardless of vitamin D status, there was no statistically significant difference in cathelicidin concentrations between subjects, consistent with the findings of other recent studies [6, 7, 12, 14, 17, 20]. Varying observations, however, have been made when determining whether a correlation between 25(OH)D and cathelicidin concentrations exists. In the literature, recent studies across a wide range of populations have demonstrated a positive correlation between 25(OH)D and cathelicidin [8–11, 15, 16, 18, 19]. Differences in age, health and initial vitamin D status between cohorts, as well as study design, may explain the conflicting data. Of note, most of the children in this study were not vitamin D deficient and the vitamin D ranges were relatively narrow. Additionally, the lack of correlation may suggest that circulating cathelicidin concentration is not reflective of what is happening at the cellular level, and in this regard, cathelicidin mRNA expression by immune cells or target tissue may be a better indicator of vitamin D’s long-term effect on the cathelicidin system.

Adipose tissue is also believed to be associated with vitamin D [26–29]. In this study, BMI and percent fat were analyzed with cathelicidin concentrations. There was no correlation between BMI at the time of the visit and cathelicidin in this population, nor was there a correlation between percent fat and cathelicidin concentration. This result is consistent with the findings of Jeng et al., who also showed no correlation with BMI and cathelicidin in adults with sepsis [9].

Although not significant, a negative trend was seen between the number of infections a child experienced and cathelicidin concentration. This suggests that cathelicidin may have a protective benefit in preventing disease, which is consistent with its antimicrobial properties. This is an exciting area of study; especially as antibiotic resistance becomes an increasing problem. Investigating ways to improve natural immune function and limit the number of infections people experience is of great importance…

In this study, a positive correlation between age and cathelicidin was shown. As individuals aged, the concentration of cathelicidin increased. Moreover, 40 subjects with samples from two subsequent years showed a highly significant (p<0.0001) increase in cathelicidin concentration from visit one to visit two. This positive correlation between cathelicidin and age was also shown in the oral environment when examining cathelicidin concentrations in saliva in healthy children aged 2 to 18 years [30]. Importantly, as Alvarez–Rodriguez and colleagues have found a negative correlation between age and immune function in healthy adult subjects, especially those over the age of 60 [15], perhaps innate immune function, in particular cytokine function, increases with age for a period of time, reaches a threshold and then decreases later in life. Interestingly, our findings that male gender correlates with cathelicidin peeks further interest in understanding gender differences in regards to the immune system. Notably, in the aforementioned study examining localized cathelicidin concentrations in saliva, the female gender was found to positively correlate with cathelicidin [30]. These findings highlight the differences one may find when examining localized and circulating levels of immune factors.

Limitations of this study include the cohort size and an unequal distribution of subjects within age groups. Of the original cohort of mothers participating in this vitamin D supplementation trial, 55% of the children were available for follow-up, and of those, 68% of those who came for follow-up (38% of the original cohort) were able to have a blood sample obtained at the time of the visit. As such, this study should be repeated with a larger sample size to replicate its findings. Inclusion of older children and young adults would allow cathelicidin to be followed throughout a longer period of childhood development and further describe how cathelicidin and innate immune function develop over time. Lastly, total circulating cathelicidin was measured rather than cell-specific mRNA expression of cathelicidin or 1,25(OH)_2_D_3_ concentration [17], which may be more closely linked with vitamin D status during fetal and childhood development.

In conclusion, this study is the first to examine cathelicidin concentrations in healthy children over time. It assists in identifying the normal ranges of circulating cathelicidin concentrations found in healthy children. It also supports the premise that cathelicidin increases with increasing age, particularly in males. With further study, cathelicidin may serve as a biomarker of enhanced immune function over time in children with age-based norms.
